# Palmitic Acid Alters Longitudinal Bone Growth While Enhancing Matrix Maturation in an Organotypic Bone Model

**DOI:** 10.3390/biom16050746

**Published:** 2026-05-19

**Authors:** Lukas Poskevicius, Victor Martin, João Gabriel Cardoso, Gintaras Juodžbalys, Pedro Sousa Gomes

**Affiliations:** 1 Faculty of Odontology, Lithuanian University of Health Sciences, A. Mickeviciaus g. 9, LT-44307 Kaunas, Lithuania; lukas.poskevicius@lsmu.lt (L.P.); gintaras.juodzbalys@lsmu.lt (G.J.); 2 BoneLab, Faculdade de Medicina Dentária, Universidade do Porto, Rua Dr. Manuel Pereira da Silva, 4200-393 Porto, Portugal; 3 LAQV/REQUIMTE, Faculdade de Medicina Dentária, Universidade do Porto, Rua Dr. Manuel Pereira da Silva, 4200-393 Porto, Portugal

**Keywords:** palmitic acid, bone, lipotoxicity, organotypic model, osteogenesis, inflammation, autophagy

## Abstract

Palmitic acid (PA), the most abundant saturated fatty acid in the human body, is implicated in lipotoxicity under hyperlipidemic conditions, with potential consequences for bone metabolism. To investigate its impact on developing bone tissue, this study used an ex vivo organotypic embryonic chick femur model, exposing femora to control (0 µM), low (50 µM), and high (200 µM) PA concentrations. A multimodal approach, integrating microtomographic, histochemical, ultrastructural, and gene expression analyses, was used to assess tissue architecture, matrix composition, mineralization, and molecular adaptations. PA exposure significantly reduced longitudinal femoral growth, as evidenced by decreased femoral length and tissue volume. Gene expression analysis revealed reduced expression of selected osteogenic differentiation-related markers, including RUNX2, BMP2, and SPP1. However, COL1A2 expression was upregulated, correlating with increased collagenous matrix deposition and enhanced mineralization in PA-treated groups. Alcian blue staining further suggested reduced proteoglycan-rich cartilage matrix, particularly at 200 µM PA. Additionally, PA modulated the expression of both pro-inflammatory and anti-inflammatory mediators, along with increased autophagy-associated responses, as suggested by the upregulation of autophagy-related genes and the presence of autophagosomes and autolysosomes. These findings indicate that PA does not simply exert a deleterious effect on bone tissue but rather redirects the developmental trajectory of the organotypic femur by reducing longitudinal growth while promoting collagen-rich matrix maturation and mineral compaction. This response may involve altered cartilage-associated endochondral processes, fatty-acid-driven metabolic adaptation, osteoblast/osteocyte maturation, and autophagy-associated matrix processing under lipid-enriched conditions.

## 1. Introduction

Hyperlipidemias encompass a broad spectrum of lipid metabolism disorders, characterized by elevated plasma lipid concentrations, including triglycerides and cholesterol. This dysregulation leads to imbalanced low-density lipoprotein (LDL) and high-density lipoprotein (HDL) levels—key mediators in lipid transport and metabolism—thereby increasing the risk of systemic pathologies [1]. Hyperlipidemias are broadly categorized as primary (familial), arising from inherited genetic disorders; or secondary (acquired), driven by environmental and physiological factors such as unbalanced diets, specific medications, and comorbid conditions like hypothyroidism or poorly controlled diabetes [2]. Hyperlipidemias are highly prevalent—affecting up to 53% of adults in the United States—and were associated with approximately 4.4 million deaths and 98.6 million disability-adjusted life years (DALYs) globally in 2019 [3,4]. Critically, they increase the risk of cardiovascular diseases, including coronary artery disease, peripheral artery disease, cerebrovascular accidents, and aneurysms, primarily driven by the pathological process of atherosclerosis [1,2].

LDL and HDL particles are composed of various lipid species, including saturated and unsaturated free fatty acids (FFAs). Among these, palmitic acid (PA; C16:O) is the most abundant saturated fatty acid in the human body [5]. FFAs are either derived from dietary intake or synthesized endogenously from carbohydrates via the fatty acid synthase pathway [5]. They serve critical functions as structural components of cellular membranes, regulators of gene expression, and substrates in energy metabolism [6]. Adipocytes, as the primary storage sites for PA, regulate their release to maintain energy balance and ensure proper metabolic function. However, excessive levels of FFAs, as seen in hyperlipidemic states, disrupt metabolic homeostasis and contribute to the development of various pathologies [7].

Recent evidence also implicates hyperlipidemia in bone metabolism dysregulation, contributing to unbalanced bone loss conditions, such as osteopenia and osteoporosis [8]. Elevated PA levels have been shown to promote osteoclastogenesis in vitro by upregulating key osteoclastogenic markers such as receptor activator of nuclear factor kappa-Β (RANK), receptor activator of nuclear factor kappa-Β ligand (RANKL), tumor necrosis factor-alpha (TNF-α), peroxisome proliferator-activated receptor-gamma (PPAR-γ), and tartrate-resistant acid phosphatase (TRAP), thus enhancing osteoclastic activity and bone resorption [9,10]. In vivo studies corroborate these findings, demonstrating that diets enriched in saturated FFAs result in bone loss and reduced biomechanical strength, largely due to enhanced osteoclastic activity and adipogenesis within the bone marrow niche [11,12]. Notwithstanding, the impact of FFAs, particularly PA, on osteoblastic function remains controversial. Some studies suggest that PA promotes apoptosis, inflammation, and inhibits osteoblast proliferation and differentiation by downregulating key osteogenic genes such as runt-related transcription factor 2 (RUNX2) and osteocalcin (OCN) [13]. In contrast, other studies suggest that PA can enhance osteoblastic differentiation through activation of pathways involving long-chain acyl-CoA synthetase-3 (ACSL3) and nuclear factor kappa-light-chain-enhancer of activated B cells (NF-κB) [14]. Moreover, the β-oxidation of FFAs has been proposed as a critical energy source for bone repair and skeletal development [8,15], suggesting a complex and potential dual role of PA in bone homeostasis.

While traditional in vitro models using osteoblastic monocultures have provided mechanistic insights, they are limited by their inability to fully replicate the complex three-dimensional architecture and native cell–cell and cell–matrix interactions inherent to bone tissue [16]. Conversely, in vivo models, though physiologically relevant, introduce systemic regulatory variables that can obscure mechanistic understanding, making it challenging to isolate the direct effects of FFAs on bone tissue [17]. To address these limitations, ex vivo organotypic models have been developed, providing a physiologically relevant microenvironment while enabling controlled experimental conditions [18]. Among these, the organotypic embryonic chick femur model provides a particularly valuable platform for studying bone dynamics in a three-dimensional context, effectively replicating the native biological environment while remaining free from systemic confounders. This model has demonstrated translational relevance to mammalian bone biology, making it a powerful tool for advancing bone research [17,19,20].

Accordingly, this study aimed to provide a comprehensive analysis of the effect of PA—the most abundant saturated fatty acid in the human body—on bone dynamics using the ex vivo organotypic embryonic chick femur model. Key parameters, including tissue structure, cellular organization, matrix mineralization, and the expression of markers associated with bone metabolism, were evaluated to elucidate the impact of PA on bone tissue functionality. This approach aims to bridge existing knowledge gaps and contribute to a more nuanced understanding of PA’s impact on skeletal health.

## 2. Methods

### 2.1. Preparation of Culture Media Containing Palmitic Acid

The selected PA concentrations were chosen to represent two levels of lipid exposure: 50 µM, a lower physiologically relevant concentration, and 200 µM, a higher lipid-enriched condition compatible with fatty-acid overload, both within ranges commonly used in experimental models of PA exposure. Culture media were prepared without (control) and with PA (P0500, Sigma-Aldrich, St. Luis, MO, USA), using ethanol, bovine serum albumin (BSA, Sigma-Aldrich, St. Luis, MO, USA), and distilled water (dH_2_O). BSA was dissolved in dH_2_O, while PA was dissolved in ethanol. Both solutions were filtered using a 0.2 μm filter (83.1826.102, Sarstedt, Nümbrecht, Germany). For the high-lipid content stock solution, the PA/ethanol mixture was gradually added to the BSA solution (5:1 molar ratio), following a previously established protocol [21], to approximate its physiological transport in plasma and to improve solubility and controlled delivery in culture medium. For the control stock solution, the same volume of ethanol without PA was added to the BSA solution. The final culture media were prepared by diluting the high-lipid content and control stock solutions into standard culture media to achieve final PA concentrations of 0 μM (control), 50 μM, and 200 μM [22]. The amount of BSA and ethanol was matched exactly across all groups, including the control, so that all media contained equivalent vehicle concentrations and differed only in PA concentration.

### 2.2. Organotypic Cultures of the Embryonic Chick Femora

Fertilized chick eggs (*Gallus domesticus*) were incubated at 37 °C with 50% relative humidity in an incubator (Octagon Advance, Brinsea, Weston-Super-Mare, UK). Embryos were euthanized on day 11 (E11) of development, and femora were carefully dissected. The femora were independently and randomly assigned to control or experimental groups before being positioned on Netwell^®^ inserts (3480, 440 μm pore diameter, Costar, Tewksbury, MA, USA), which were placed into six-well plates containing 1 mL of culture medium. The medium consisted of α-MEM (Gibco, Dublin, Ireland) supplemented with 1% (*v*/*v*) 2-phospho-L-ascorbic acid and 1% penicillin/streptomycin/amphotericin-B (all reagents from Sigma-Aldrich, St. Luis, MO, USA). Femora were cultured at the air/liquid interface at 37 °C in a humidified atmosphere containing 5% CO_2_, with medium changes performed daily. PA exposure was initiated after the initial 6-day culture period to allow the embryonic femora to stabilize under organotypic culture conditions and to preserve ongoing tissue growth and matrix organization before experimental treatment. After 6 days of incubation, the culture medium was replaced with media containing PA at 0, 50, and 200 μM for an additional 5 days, under the described conditions. At the end of the 11-day experimental period, femora were either snap-frozen in liquid nitrogen or fixed in 70% ethanol or 4% paraformaldehyde (PFA), according to the subsequent characterization. Each femur constituted a biological replicate. As the use of avian embryos during the first two-thirds of development for research purposes is not regulated under European (Directive 2010/63/EU) or national (Decreto-Lei no. 113/2013) legislation, no specific regulatory approval was required for the experimental procedures.

#### 2.2.1. Bone Length

Femoral growth was evaluated by photodocumenting the femora at day 6, prior to the exposure to PA, and at day 11, at the end of the experiment. Length changes were measured using ImageJ software (version 1.53k.2.3.2), with growth calculated as the difference between the two time points. Analyses were performed in quintuplicate.

#### 2.2.2. Microtomographic Evaluation (µCT)

Fixed femora were scanned using a Skyscan 1276 System (Bruker, Kontich, Belgium) at 40 kV, 100 μA, with an exposure time of 800 ms and a voxel size of 4.5 μm. Projection images were reconstructed with NRecon software (version 1.7.4.2, Bruker, Kontich, Belgium). Then, samples were oriented along the sagittal axis using DataViewer software (version 1.5.6.2, Bruker, Kontich, Belgium). Morphological analysis of the whole bone was carried out using CTAnalyser software (version 1.17.7.2, Bruker, Kontich, Belgium), assessing histomorphometric parameters such as bone volume (BV), tissue volume (TV), bone surface (BS), and bone mineral density (BMD), as previously described [23]. Three-dimensional images were generated using CTVox software (version 3.3.0, Bruker, Kontich, Belgium). Samples were analyzed in quintuplicates.

#### 2.2.3. Histochemical and Histomorphometric Evaluation

Fixed femora were processed according to a previously described protocol [18]. Briefly, samples were embedded in paraffin blocks, cut into 5 μm-thick sections, and stained using a combination of Alcian blue and Picrosirius red solutions (Sigma-Aldrich, St. Luis, MO, USA) to visualize glycosaminoglycans (blue) and collagenous matrix (red), respectively. Moreover, Oil red-O staining (CI 26125, Sigma-Aldrich, St. Luis, MO, USA) was performed to detect hydrophobic lipids, while excluding polar lipids from the cell membranes [24]. Mineralized bone was identified by Von Kossa staining (Silver plating kit, Sigma-Aldrich, St. Luis, MO, USA), with calcified tissue appearing dark brown to black. Images were captured using a Zeiss Axiolab 5 microscope equipped with an Axiocam 5 Color Camera (Zeiss, Oberkochen, Germany). The areas of cartilage, collagen, and mineralized content were quantified in ImageJ (version 1.53k.2.3.2) by segmentation at mid-diaphysis, using Otsu’s thresholding algorithm [18]. All assays were performed in quintuplicates.

#### 2.2.4. Transmission Electron Microscope (TEM) Observation

For ultrastructural analysis, femora were fixed and subjected to a graded ethanol dehydration series. Then, samples were embedded in Spurr’s resin, sectioned into ultrathin slices (35–50 nm), and mounted on TEM grids (Agar Scientific, Stansted, UK) for observation. Observations were performed using a JEM 1400 microscope (JEOL, Tokyo, Japan). The assay was performed in triplicate.

#### 2.2.5. Gene Expression Analysis

Frozen femora were lysed in TRIzol reagent (Invitrogen, San Diego, CA, USA), and total RNA was isolated from DNA and proteins following the established manufacturer’s protocol. RNA concentration and purity were determined by absorbance reading (260/280 nm) using a Take3 microplate module (Gen5, BioTek) and a microplate reader (Synergy HT; BioTek, Winooski, VT, USA). Further, the conversion to cDNA was performed using a two-step reverse transcription kit (NZY Kit, NZYTECH, Lisbon, Portugal). Quantitative PCR was performed using the CFX384 real-time PCR system (Bio-Rad, Hercules, CA, USA), with the iTaq Universal SYBR green Supermix (Bio-Rad, Hercules, CA, USA) and gene-specific primers (Table 1). Gene expression was normalized to the housekeeping gene GAPDH, and relative quantification was calculated using the 2^–ΔΔCt^ method. The assay was performed in triplicate.

### 2.3. Statistical Analysis

Statistical analysis was performed using IBM SPSS software v.27 (IBM Corp., Armonk, NY, USA). Data normality was assessed using the Shapiro–Wilk test. For variables that demonstrated normality, a one-way ANOVA followed by Tukey’s multiple-comparison test was used. Non-parametric datasets were analyzed using the Kruskal–Wallis test. The level of significance was set at *p* ≤ 0.05. Each femur constituted a biological replicate.

## 3. Results

Embryonic chick femora, isolated at day 11 of development (E11), were cultured at the air–liquid interface for 11 days, with palmitic acid (PA) introduced during the final 5 days. The impacts of PA on femoral growth, structure, tissue organization, and gene expression were systematically evaluated.

**Figure 1:**
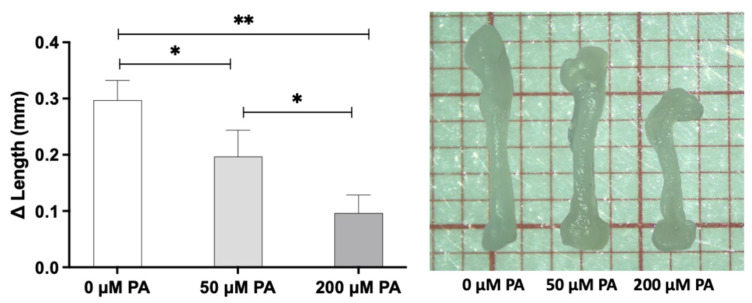
Left—Length variation (Δ Length) between the initial and final measurements of femora. * Significantly different between conditions—*p* ≤ 0.05; ** *p* ≤ 0.01. Data were analyzed using one-way ANOVA followed by Tukey’s post hoc test (*n* = 5). Right—Representative images of femora at final measurement. The scale on the graph paper corresponds to 1 mm.

Morphometric analysis revealed a concentration-dependent effect of PA on femoral growth (Figure 1). In the control group (0 µM PA), femoral length increased by approximately 0.3 mm over the culture period. Exposure to 50 µM PA resulted in a moderate reduction in length variation, while 200 µM PA significantly impaired longitudinal bone growth. Additionally, femora exposed to PA, particularly at 200 µM, exhibited atypical epiphyseal curvature, suggesting potential macrostructural alterations.

**Figure 2:**
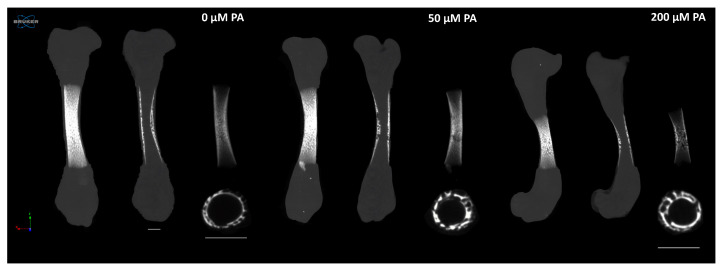
Representative 2D and 3D microtomographic reconstructions of femora. The sequence of images for each group progresses from maximum-intensity projection, sagittal section, segmented mineralized tissue, and a cross-section of the central diaphysis (scale bars correspond to 500 μm).

**Figure 3:**
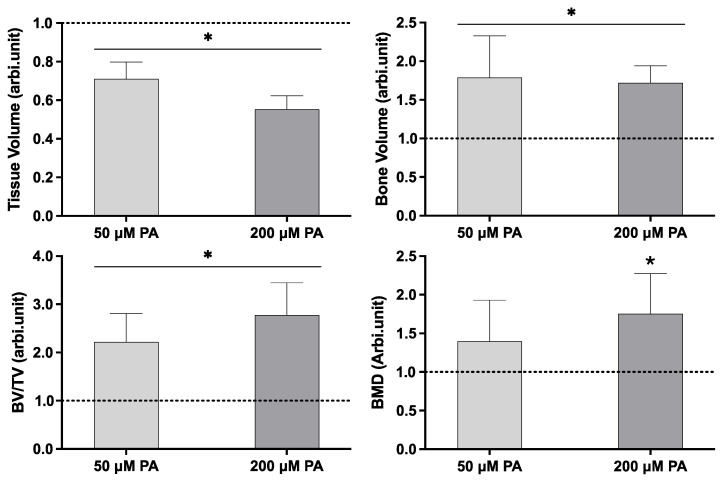
Histomorphometric indices from microtomographic evaluation, normalized to control (0 µM PA, set as 1.0), represented by the dashed line. * Significantly different from control, *p* ≤ 0.05. Data were analyzed using one-way ANOVA followed by Tukey’s post hoc test (*n* = 5).

Microtomographic analysis provided a detailed structural and histomorphometric assessment of femoral structure (Figure 2 and Figure 3). In the control group (0 µM PA), femora exhibited longer bones with well-defined mineralized regions extending from the central diaphysis into the metaphysis. In contrast, PA-treated femora showed a dose-dependent reduction in bone length (Figure 2).

Quantitative histomorphometric analysis (Figure 3) revealed that both PA-treated groups had significantly lower tissue volume (TV) than the control group. Despite this reduction, bone volume (BV) and the BV/TV ratio were significantly increased in the PA-exposed femora. Bone mineral density (BMD) increased in PA-treated groups, with the 200 µM PA group showing a significant increase compared to the control.

**Figure 4:**
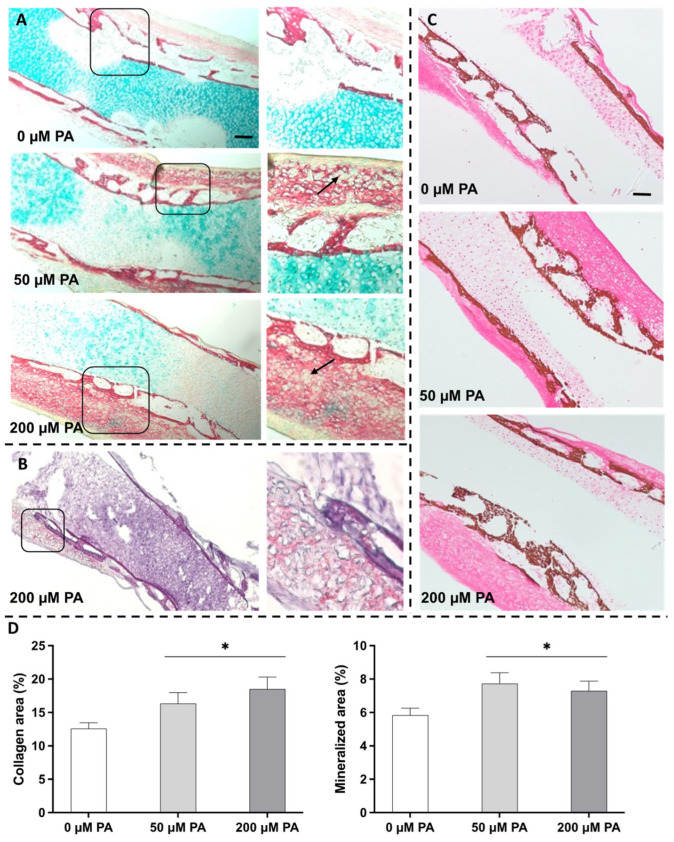
Representative micrographs of histochemical staining of femora. (**A**) Alcian blue and Sirius red (AB/SR) staining for proteoglycans and collagenous tissue, respectively. Black arrows correspond to areas of potential lipid accumulation. (**B**) Oil Red-O staining evidencing lipid incorporation (in red) in the outer diaphysis layer. (**C**) Von Kossa (VK) staining for mineralized tissue identification. (**D**) Quantitative analysis of collagenous and mineralized areas. Scale bars correspond to 100 μm. * Significantly different from control, *p* ≤ 0.05. Data were analyzed using one-way ANOVA followed by Tukey’s post hoc test (*n* = 3).

Histochemical staining (Figure 4) provided insights into tissue organization and matrix composition. In the control group, a collagen-rich outer layer displayed a progressive centripetal trabecular organization, surrounding an inner proteoglycan-rich layer—features characteristic of active osteogenesis (Figure 4A). Although PA exposure did not disrupt the overall tissue organization, notable structural changes were observed. The collagenous outer layer showed increased thickness, with intensified Sirius red staining, indicating enhanced collagen deposition. In parallel, the Alcian blue-positive proteoglycan-rich inner region appeared less extensive and showed reduced staining intensity in PA-treated samples, particularly in the 200 µM PA group. Additionally, the trabecular structure appeared more compact and condensed. These alterations point to a shift in the bone’s structural arrangement and matrix composition.

PA-treated groups exhibited rounded, whitish regions within the collagenous outer layer, indicative of potential lipid accumulation. This was supported by Oil Red-O staining (Figure 4B), which confirmed the presence of lipids in these regions, particularly in the marginal diaphyseal region, substantiating lipid incorporation into the bone matrix. Additionally, von Kossa staining (Figure 4C) further revealed a more compacted organization of the mineralized trabeculae in PA-treated samples. Histomorphometric analyses corroborated these observations, demonstrating significant increases in both collagenous and mineralized areas in the PA-treated groups (Figure 4D).

**Figure 5:**
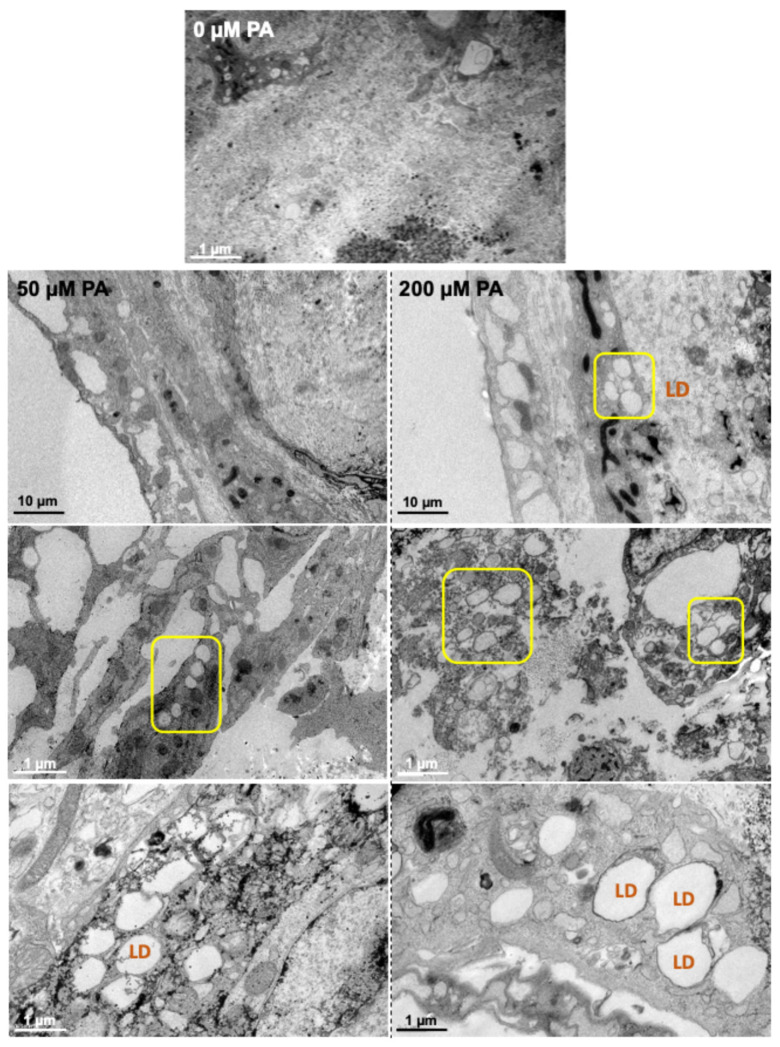
Representative TEM images of femora. Lipid droplets (LDs) are visible in both 50 and 200 µM PA-treated groups, highlighted in yellow squares.

Ultrastructure exploration of the femora was conducted using TEM (Figure 5). The control group (0 µM PA) exhibited a uniform, dense outer layer with a relatively smooth and light appearance. In contrast, PA-treated groups exhibited a thinner and less compact outer layer, with numerous empty spaces. Additionally, vesicles, corresponding to lipid droplets (LDs), were broadly abundant in the femoral core of both PA-treated groups, particularly in the 200 µM PA group, indicating intracellular lipid accumulation and matrix disruption.

**Figure 6:**
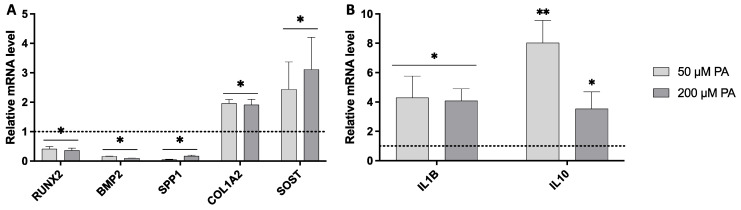
Relative expressions of (**A**) osteogenic and (**B**) inflammation-related genes in femora. GAPDH was used as the housekeeping gene, with control expression levels normalized to 1.0 (dashed line). Significantly different from control (0 µM PA); * *p* ≤ 0.05; ** *p* ≤ 0.01). Data were analyzed using one-way ANOVA followed by Tukey’s post hoc test (*n* = 3).

Gene expression analysis revealed that PA exposure induced significant alterations in key osteogenic and inflammatory markers (Figure 6). The expression of *RUNX2*, a critical transcription factor driving osteogenic differentiation, was significantly downregulated in both PA-treated groups compared with the control, indicating attenuation of selected osteogenic differentiation-related signaling pathways. This reduction was accompanied by a substantial decrease (around 80%) in the expression of other osteogenic differentiation-related markers, including *BMP2* and *SPP1*, associated with osteoblastic differentiation and matrix maturation. In contrast, *COL1A2*, which encodes the alpha-2 chain of type I collagen, was significantly upregulated in both PA-treated groups, showing an approximate 2-fold increase. Notably, SOST, a marker associated with Wnt signaling regulation and late osteoblast/osteocyte maturation, was also significantly upregulated. Regarding inflammation-related markers, the pro-inflammatory gene *IL1B* was markedly upregulated in both PA-treated groups, exhibiting an approximate 4-fold increase compared to the control. In parallel, the anti-inflammatory gene *IL-10* was also significantly upregulated, with expression levels increasing by approximately 4- to 8-fold.

**Figure 7:**
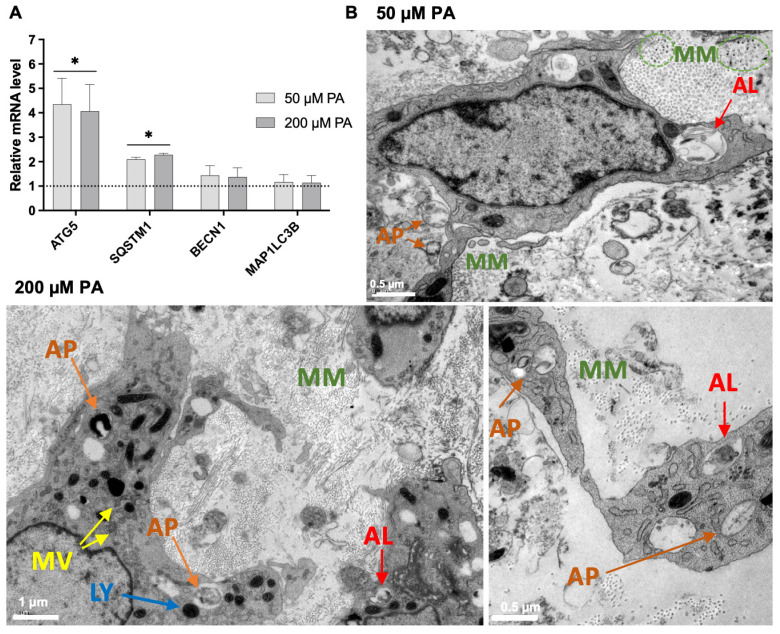
(**A**)—Relative expressions of genes related to autophagy. GAPDH was used as the housekeeping gene, with expression levels in the control group normalized to 1.0 (dashed line). * Significantly different from control (0 µM PA). Data were analyzed using one-way ANOVA followed by Tukey’s post hoc test (*n* = 3). (**B**)—Representative TEM images of femora showing autophagy-associated ultrastructural features in PA-exposed groups (AP—Autophagosome; LY—Lysosome; MM—Mineralizing matrix; AL—Autolysosome; MV—Mineralized vesicle).

Autophagy-related gene expression and TEM analysis suggested increased autophagy-associated activity in PA-exposed femora (Figure 7). Significant upregulation of ATG5 and SQSTM1 was observed in both PA-treated groups (Figure 7A). TEM images revealed the presence of autophagosomes and autolysosomes in both the 50 and 200 µM PA-treated groups. Additionally, the progressive mineralization of the collagenous matrix was evident under both PA conditions (Figure 7B), as evidenced by electron-dense mineral deposits within the matrix.

## 4. Discussion

Lipids play a central role in bone tissue metabolism, modulating the functionality of both osteoclasts and osteoblast-lineage cells [8,12]. While the contribution of lipid metabolism to osteoclast differentiation and activity is well established [12], the effects of fatty acids on osteoblastic functionality are more complex and appear to depend on concentration, exposure duration, cellular maturation stage, and metabolic context. Some studies suggest that FFAs impair osteoblastic proliferation and differentiation [13], whereas others propose that lipids contribute to supporting the energetic requirements of osteoblast differentiation, matrix synthesis, and mineralization [25,26].

This study utilized an organotypic bone model to explore the effects of palmitic acid (PA)—the most abundant saturated fatty acid in the human body—on bone tissue dynamics, an analysis that, to the best of the authors’ knowledge, is unprecedented. This model preserves the 3D architecture and complex cellular interactions of bone tissue while eliminating systemic confounders [17,27]. PA was tested at physiologically relevant concentrations—50 µM (low) and 200 µM (high)—bound to BSA in a 5:1 molar ratio, replicating its natural transport in plasma [21].

Our findings revealed that PA exposure significantly impaired longitudinal femoral growth in a concentration-dependent manner, as evidenced by reduced femoral length and tissue volume (Figure 1 and Figure 3). This effect indicates reduced tissue expansion, which may be related to lipid-induced cellular stress affecting cell-cycle regulation, viability, and proliferation [24,28]. Excess PA can overwhelm metabolic pathways, leading to mitochondrial dysfunction [21,29], increased reactive oxygen species (ROS) production, and the accumulation of misfolded proteins [30,31], all of which may compromise cell viability and proliferation. TEM analysis confirmed substantial intracellular lipid accumulation (Figure 5), while histochemical staining evidenced lipid deposits within bone matrix (Figure 4B).

In addition to direct effects on osteoblast-lineage cells, PA may have affected the cartilaginous component of the developing femur. Longitudinal growth of embryonic long bones is primarily driven by endochondral processes involving chondrocyte proliferation, maturation, hypertrophy, and matrix remodeling. Therefore, the reduced femoral elongation observed in PA-treated samples may also reflect altered chondrocyte dynamics, rather than being solely attributable to changes in osteoblast activity. In support of this interpretation, altered fatty-acid availability has been shown to affect cartilage formation during skeletal repair [32], and recent evidence indicates that altered fatty acid metabolism in chondrocytes can impair SOX9 stability through ubiquitination-mediated degradation, thereby affecting chondrocyte differentiation and cartilage homeostasis [33]. Although SOX9 expression and other chondrocyte-specific markers were not evaluated in the present study, the observed reduction in tissue volume and femoral length, together with the apparent reduction and decreased staining intensity of the proteoglycan-rich region, suggests that PA exposure may have interfered with endochondral growth mechanisms. This aspect warrants further investigation, particularly through the assessment of chondrogenic markers such as SOX9, COL2A1, ACAN, COL10A1, and MMP13.

In parallel, the expression of selected osteogenic differentiation-related markers was significantly reduced, as evidenced by the downregulation of *RUNX2*, a master regulator of osteogenesis, alongside reduced expressions of *BMP2* and *SPP1*, markers associated with osteoblastic differentiation and matrix maturation [34]. This transcriptional profile is consistent with previous reports describing PA-mediated attenuation of osteoblast differentiation-related markers in both in vitro [35,36] and in vivo [37] systems. SOST expression was also significantly upregulated. While sclerostin is classically recognized as an inhibitor of canonical Wnt signaling and bone formation [31], SOST is also associated with late osteoblast/preosteocyte differentiation and osteocyte maturation [38]. Therefore, in the context of the present organotypic femur model, SOST upregulation may reflect changes in the maturation status of osteoblast-lineage cells, including progression toward a late osteoblast/preosteocyte or osteocyte-like phenotype, rather than exclusively reinforcing osteogenic inhibition.

Interestingly, despite the attenuation of selected osteogenic differentiation-related markers, both microtomographic and histochemical analyses revealed increased matrix mineralization in PA-treated groups (Figure 2, Figure 3 and Figure 4C). This observation suggests that PA exposure differentially affects longitudinal growth, osteogenic gene expression, and matrix maturation. One key factor appears to be the increased collagen deposition, as evidenced by elevated *COL1A2* expression and intensified Sirius red staining (Figure 4A). While collagen accumulation and mineralization are typically integral components of osteogenic responses, increased collagen deposition may also occur as part of a stress-associated extracellular matrix remodeling response. In this regard, PA has been linked to pro-fibrotic matrix remodeling in non-skeletal tissues, partly through mechanisms involving metabolic stress, endoplasmic reticulum stress, inflammatory signaling, and altered collagen turnover [39,40]. Therefore, in the present model, PA-induced lipid accumulation and associated cellular stress responses may have favored COL1A2 upregulation and collagen-rich matrix deposition, even in the context of reduced expression of selected osteogenic differentiation-related genes. This expanded collagenous matrix may then have acted as a structural scaffold for mineral nucleation and deposition, contributing to the increased mineralized area and BMD observed in PA-treated groups.

Inflammatory signaling may have also contributed to the altered growth and matrix phenotype observed in PA-treated femora. PA is known to activate Toll-like receptors, including TLR-2 and TLR-4, in bone-related cells [41], triggering NF-kB signaling, inflammasome activation, and the production of pro-inflammatory cytokines, particularly interleukin-1 beta (IL-1β) [41]. Consistent with this mechanism, IL1B was markedly upregulated in PA-exposed groups. IL-1β can modulate osteoblast-lineage cell function by suppressing differentiation-related regulators such as RUNX2 [13,36] and by promoting cellular stress or apoptosis under lipotoxic conditions [42]. These effects may have contributed to the reduced femoral elongation and tissue volume observed in the present study. Furthermore, IL-1β has been reported to upregulate SOST expression [43], suggesting that PA-induced inflammatory signaling may also participate in the modulation of Wnt-associated bone regulatory pathways.

Concurrently, a significant upregulation of IL-10, a key anti-inflammatory cytokine, was observed in PA-treated samples, with expression increasing approximately 8-fold at the lower PA concentration. IL-10 counteracts pro-inflammatory signaling by inhibiting pro-inflammatory cytokine production and promoting tissue homeostasis [44,45]. Its upregulation in PA-treated samples may therefore represent a compensatory response aimed at limiting inflammation-associated tissue stress. Notably, IL-10 has also been associated with extracellular matrix regulation and collagen deposition in inflammatory environments [46,47], as well as with osteoblast differentiation-related responses [44]. Thus, although IL-10 upregulation should not be interpreted as the sole mechanism underlying the increased collagenous matrix, it may have contributed to the broader matrix-remodeling response observed in PA-treated femora.

Autophagy may represent an additional adaptive response to PA-induced metabolic and lipid-associated stress. Acting as a cellular defense mechanism, autophagy contributes to the removal of damaged organelles, regulation of protein quality control, and maintenance of extracellular matrix homeostasis under stress conditions [48]. PA exposure has been shown to induce autophagy in osteoblastic cells, leading to increased autophagosome formation and the upregulation of autophagy-related genes such as ATG5 and SQSTM1 [42], findings that are consistent with the present study. Beyond its cytoprotective role, autophagy has also been implicated in type I collagen biosynthesis and matrix organization [49,50]. For instance, inhibition of ATG5 has been shown to suppress COL1A1 expression, likely due to the accumulation of misfolded collagen [51], while SQSTM1/p62-related mechanisms have been associated with increased extracellular matrix deposition of collagen [52]. Moreover, given the role of SQSTM1/p62 in bone-cell homeostasis and its reported involvement in Ajuba regulation [53], p62–Ajuba-related autophagy signaling may represent an additional contributory pathway linking PA-induced lipid stress to the altered bone-cell and matrix responses observed here. Therefore, the increased expression of autophagy-related genes observed here may have contributed to the maintenance of collagen processing and matrix organization under PA exposure, although a direct causal relationship between autophagy activation and increased collagen deposition cannot be established from the present data.

Autophagy has also been implicated in matrix mineralization, although its role in the present model should be interpreted cautiously. Deletion of ATG5 in osteoblasts has been shown to reduce bone mass and impair mineralized nodule formation in both in vitro and in vivo models [54,55]. Likewise, p62, the product of SQSTM1, has been associated with increased osteoblastic mineralization [56]. Mineral formation is thought to be initiated within intracellular vesicles, which act as nucleation sites for apatite crystals before their extracellular release [57]. Emerging evidence indicates that autophagic vacuoles may function as transport carriers for these mineral precursors, ensuring their efficient delivery and deposition into the ECM [58]. In the present study, the upregulation of ATG5 and SQSTM1, together with the ultrastructural detection of autophagosomes, autolysosomes, mineralizing matrix, and mineralized vesicles, is compatible with a possible contribution of autophagy-associated vesicular processing to mineral deposition. However, these findings remain correlative, and further mechanistic studies would be required to determine whether autophagy directly mediates the increased mineralized matrix observed in PA-treated femora.

A limitation of the present study is that the ex vivo embryonic chick femur model does not fully reproduce the systemic regulation present in vivo, including circulating lipid metabolism, endocrine signaling, immune responses, vascularization, and whole-organism bone remodeling. Therefore, the findings should be interpreted primarily in the context of developmental/endochondral bone growth and matrix maturation, rather than adult skeletal remodeling. Future in vivo studies will be needed to validate these observations under systemic metabolic conditions.

## 5. Conclusions

Collectively, these findings indicate that PA exposure alters developmental bone dynamics in the organotypic embryonic femur model. PA reduced femoral elongation and tissue volume, suggesting impaired longitudinal growth, while simultaneously increasing collagen deposition, mineralized matrix accumulation, and mineral density. The apparent reduction in the Alcian blue-positive proteoglycan-rich region further suggests that cartilage-associated endochondral processes may contribute to this phenotype. Thus, PA should not be interpreted as inducing a generalized impairment of bone formation, but rather as differentially affecting longitudinal growth, matrix organization, and mineral maturation. Future studies should clarify the contributions of chondrocyte differentiation, osteoblast-lineage maturation, lipid handling, autophagy-associated vesicular processing, and osteoclast/chondroclast-related resorptive activity to these effects.

## Figures and Tables

**Table 1:** Primers used for amplifying the targeted genes.

Gene	Unique Assay ID	Gene	Unique Assay ID
*GAPDH*	qGgaCED0029996	*IL1B*	qGgaCED0028444
*RUNX2*	qGgaCID0019198	*IL10*	qGgaCED0029223
*BMP2*	qGgaCID0027472	*ATG5*	qGgaCED0030946
*SPP1*	qGgaCED0023869	*SQSTM1*	qGgaCED0026040
*COL1A2*	qGgaCED0025365	*BECN1*	qGgaCED0024443
*SOST*	qGgaCED0029174	*MAP1LC3B*	qGgaCED0022681

Abbreviations: *GAPDH*—Glyceraldehyde-3-Phosphate Dehydrogenase; *RUNX2*—Runt-Related Transcription Factor 2; *BMP2*—Bone Morphogenetic Protein 2; *SPP1*—Secreted Phosphoprotein 1; *COL1A2*—Collagen Type I Alpha 2; *SOST*—Sclerostin; *IL1B*—Interleukin 1 beta; *IL10*—Interleukin 10. *MAP1LC3B*—Microtubule-associated proteins 1A/1B light chain 3B; *ATG5*—Autophagy protein 5; *SQSTM1*—Sequestosome 1; *BECN1*—Beclin-1.

## Data Availability

The raw data supporting the conclusions of this article will be made available by the authors on request.
